# Changes in the Place of Death of Patients With Cancer After the Introduction of Insurance-Covered, Home-Based Hospice Care in Korea

**DOI:** 10.1001/jamanetworkopen.2023.41422

**Published:** 2023-11-06

**Authors:** Il Yun, Sung-In Jang, Eun-Cheol Park, Suk-Yong Jang

**Affiliations:** 1 Department of Public Health, Graduate School, Yonsei University, Seoul, Republic of Korea; 2 Institute of Health Services Research, Yonsei University, Seoul, Republic of Korea; 3 Department of Preventive Medicine, Yonsei University College of Medicine, Seoul, Republic of Korea; 4 Department of Healthcare Management, Graduate School of Public Health, Yonsei University, Seoul, Republic of Korea

## Abstract

**Importance:**

Although hospice care has been covered by health insurance for the purpose of improving the quality of life of patients with terminal cancer as well as their caregivers, few studies have evaluated the outcomes of the policy to cover home-based hospice care services.

**Objective:**

To investigate the changes in the place of death of patients with cancer after the introduction of insurance-covered, home-based hospice care services in Korea.

**Design, Setting, and Participants:**

This cohort study used data from February 1, 2018, to December 31, 2021, from the Causes of Death Statistics database, released annually by Statistics Korea, which contains information on all deaths in the country. Individuals who died of cancer, a representative hospice-eligible disease, were assigned to the case group, and those who died of dementia, a non–hospice-eligible disease, were assigned to the control group. A total of 218 522 individuals constituted the study population.

**Exposure:**

Because the Korean Health Insurance Service had begun covering home-based hospice care services on September 1, 2020, and the last follow-up date was December 31, 2021, the follow-up periods for before and after intervention were 31 months and 16 months, respectively (preintervention period: February 1, 2018, to August 31, 2020; postintervention period: September 1, 2020 to December 31, 2021).

**Main Outcomes and Measures:**

The place of death was categorized as a binary variable according to whether it was the person’s own home or not. Comparative interrupted time-series models with segmented regression were applied to analyze the time trend and its change in outcomes.

**Results:**

Of the 218 522 deaths eligible for the analysis (mean [SD] age at death, 78.6 [8.8] years; 130 435 men [59.7%]), 207 459 were due to cancer, and 11 063 were due to dementia. Immediately after the introduction of home-based hospice care, the rate of home deaths was 24.5% higher for patients with cancer than for those with dementia (estimate, 1.245 [95% CI, 1.030-1.504]; *P* = .02). The difference in the level change between cancer deaths and dementia deaths, on intervention, was more pronounced for those living in rural areas (estimate, 1.320 [95% CI, 1.118-1.558]; *P* = .001). In addition, a higher educational level was associated with a larger difference in the immediate effect size due to home-based hospice care (low educational level: estimate, 1.205 [95% CI, 1.025-1.416]; *P* = .02; middle educational level: estimate, 1.307 [95% CI, 0.987-1.730], *P* = .06; high educational level: estimate, 1.716 [95% CI, 0.932-3.159]; *P* = .08).

**Conclusions and Relevance:**

In this cohort study exploring the changes in the place of death for patients with cancer after the insurance mandates for home-based hospice care in Korea, the probability of patients with cancer dying in their own homes increased after the intervention. This finding suggests the need to broaden the extent of home-based hospice care to honor the autonomy of individuals with terminal illness and improve their quality of death.

## Key Points

**Question:**

Is health insurance coverage for home-based hospice care associated with changes in the place of death among Korean patients with cancer?

**Findings:**

This cohort study of 218 522 individuals identified that hospice-eligible patients with cancer were more likely than patients with dementia to die at home after home-based hospice care was covered by health insurance in Korea.

**Meaning:**

Considering that many patients with terminal illness want a dignified death at home with palliative care, this study suggests that home-based hospice care is associated with improved quality of life at the end of life.

## Teaser

This cohort study investigates the changes in the place of death of patients with cancer after the introduction of insurance-covered, home-based hospice care services in Korea.

## Introduction

Over the past few decades, cancer has become the leading cause of death worldwide. As of 2020, approximately 10 million people died of cancer, representing approximately one-sixth of all deaths.^1^ Despite advancements in cancer diagnosis and treatment that have resulted in longer lifespans for patients with cancer, many patients still receive a diagnosis at the terminal stage. Patients with advanced cancer may experience physical and psychological symptoms due to their disease, treatment, or other comorbidities.^2^ Symptoms are often not addressed with conventional care, leading to a significant effect on the patients’ quality of life and family relationships.^3^ Against this background, hospice and palliative care programs were introduced to improve the quality of life of patients with terminal illness and their caregivers by providing relief rather than a cure.

Hospice care and palliative care are essential aspects of integrated patient-focused health care. It is a global and ethical responsibility to alleviate severe health-related pain, be it physical, psychological, or spiritual.^4^ Hospice care services can be provided in a variety of settings, such as hospitals, nursing homes, and patients’ own homes,^5^ depending on the health care system in each country. The World Health Organization has estimated that approximately 56.8 million individuals, including 25.7 million in the last year of life, require palliative care each year and that there is a growing need for hospice care services due to the aging population and the increase in the prevalence of chronic diseases, such as cancer, heart disease, and dementia.^6,7^ However, only approximately 14% of those in need of hospice care are currently receiving it.^6,7^ Similarly, discussions on dying well and the improvement of the right to self-determination at the end of life are being actively conducted in Korea owing to the rapid aging of the population. In Korea, 1 in 4 deaths is due to cancer, and approximately 23.2% of all cancer-related deaths involve hospice care services.^8^ However, most patients use hospital-based hospices, and only 4% of all hospice users used home-based hospices,^8^ which raises doubts about the effectiveness of the policy.

Several studies have previously evaluated the association of hospice use with medical applications and expenses before the death of patients with terminal cancer. They reported that providing hospice care at an earlier stage could potentially reduce unnecessary hospital admissions, use of health care services,^9^ and medical expenditures.^10^ Because it has been only 3 years since the insurance mandate for home-based hospice care in Korea, only a few studies have been conducted to evaluate its effectiveness. Patients who opt for home-based hospice care are provided with palliative care in their own homes and die in the comfort of their homes. However, to our knowledge, no study has yet evaluated whether the policy was implemented as intended.

Therefore, the present study aimed to explore the changes in the place of death of patients with cancer after the introduction of home-based hospice care in Korea. We hypothesized that the intervention would increase the likelihood of patients with cancer dying at home compared with patients with dementia.

## Methods

### Data and Study Population

This retrospective cohort study used data from the Causes of Death Statistics database released annually by Statistics Korea. This database includes information on the sociodemographic characteristics and mortality of all individuals who have died in Korea. The date, time, and place of death of all the deceased individuals were recorded, and the cause of death was coded according to the clinically determined *International Statistical Classification of Diseases and Related Health Problems, Tenth Revision* (*ICD-10*). The study protocol was approved by the institutional review board of Severance Hospital, Yonsei University Health System, which waived the requirement for informed consent because the Causes of Death Statistics did not contain any identifiable information. Furthermore, ethical approval for the use of data was not required because Statistics Korea provides publicly accessible data. In conducting this observational study, we adhered to the Strengthening the Reporting of Observational Studies in Epidemiology (STROBE) reporting guideline designed for cohort studies.

The present study included data from February 1, 2018, when the Act on Hospice and Palliative Care and Decisions on Life-Sustaining Treatment for Patients at the End of Life was implemented in Korea, to December 31, 2021. Those who died of cancer were classified into the case group, those who died of dementia were classified into the control group, and those who died before the age of 65 years were excluded. A total of 218 522 individuals constituted the study population and were eligible for the analysis.

### Variables

The main variable of interest was the time of the introduction of home-based hospice care in Korea, which divided the time span studied. Because the Korean Health Insurance Service began providing health insurance coverage for home-based hospice services from September 1, 2020, and the last follow-up date was December 31, 2021, we determined the follow-up periods for before and after intervention as 31 months and 16 months, respectively. The preintervention period was from February 1, 2018, to August 31, 2020, and the postintervention period was from September 1, 2020, to December 31, 2021. We set deaths due to cancer, which account for most hospice users, as the case group (*ICD-10* codes C00-D49) and deaths due to dementia (*ICD-10* codes F00-F03, G30, and G311),^11^ which is not yet eligible for hospice care in Korea but is significantly prevalent and is a major cause of mortality,^12,13^ as the control group.

The outcome variable was the place of death, which was categorized as a binary variable (ie, whether it was the person’s own home or not). As covariates, we included sociodemographic factors, such as sex (men and women), age at death (65-69, 70-74, 75-79, and ≥80 years), residential area (urban and rural), occupation (white collar [professional, managerial, or administrative workers], blue collar [manual or industrial workers], pink collar [service-oriented workers], and other), marital status (married and single), and educational level (low [elementary school graduation or lower], middle [high school graduation or lower], and high [college graduation or higher]).

### Statistical Analysis

Descriptive statistics are presented as frequencies and percentages. Subsequently, to evaluate the association of the policy with the time trend and the change in outcomes, we applied comparative interrupted time-series (CITS) models. In a CITS design, the evaluation of policy associations involves comparing the degree of deviation from the baseline trend between the case and control groups to assess whether the deviation in the case group is greater.^14^ The CITS was modeled using a linear regression model that included 7 time-related variables. Because we used the log-link function in the generalized linear model to conduct the segmented regression, converting the model coefficient exponentials (exp) to show the trends and changes in outcomes on the original scale was necessary. Therefore, to interpret model coefficients, we had to convert log [*E*(*Y_i_*)] into multiplicative interpretations for the original scale, which is represented as *E*(*Y_i_*) = *μ_i_*: log (*μ_i_*) = β_0_ + β_1_*T* + β_2_ + β_3_*XT* + β_4_*Z* + β_5_*ZT* + β_6_*ZX* + β_7_*ZXT* + *e*.

In this regression equation, *T* represents time, shown on a monthly basis. *X* indicates whether the intervention had been introduced yet, and *XT* indicates the time after the intervention. *Z* is a binary variable that distinguishes the case group from the control group. *ZT* represents the time for the case group, *ZX* refers to the study phase for the case group, and *ZXT* represents the time after interruption for the case group. Therefore, *ZT*, *ZX*, and *ZXT* were all assigned 0 for the control group. Regarding the regression coefficients in this model, intercept β_0_ estimates the baseline level of the outcome; β_1_ estimates the preintervention trend of the outcome for the control group; β_2_ estimates the level change after the intervention for the control group, indicating the immediate effect size of the intervention for the control group; and β_3_ estimates the slope change after the intervention for the control group. Intercept β_4_ estimates the level difference between the case group and the control group before intervention, β_5_ estimates the difference in slope between the case group and the control group before intervention, and β_6_ estimates the difference in absolute level change between the case group and the control group after intervention. Finally, β_7_ estimates the difference in slope change before and after the intervention between the case group and the control group.^15^

All statistical analyses were conducted using SAS software, version 9.4 (SAS Institute Inc). The results are presented as parameter estimates, SEs, 95% CIs, and *P* values, where statistical significance was determined by 2-sided tests and *P* < .05 considered significant.

## Results

A total of 218 522 individuals (mean [SD] age at death, 78.6 [8.8] years; 130 435 men [59.7%] and 88 087 women [40.3%]) were eligible for the present analysis (144 506 before the introduction of the intervention and 74 016 after the introduction of the intervention). Men (130 435 [59.7%]), people who died at 80 years of age or older (100 225 [45.9%]), rural residents (130 581 [59.8%]), and those who were single (123 894 [56.7%]) were the most frequently reported among all participants (Table 1). Subsequently, 207 459 deaths were due to cancer (classified as the case group), and 11 063 deaths were due to dementia (classified as the control group) (Table 2). A total of 20 851 cancer deaths (10.1%) and 3246 dementia deaths (29.3%) occurred at home.

**Table 1.** Characteristics of the Study Population

Variable	Patients receiving insurance-covered home-based hospice care, No. (%)	
	Preintervention period (February 2018 to August 2020) (n = 144 506)	Postintervention period (September 2020 to December 2021) (n = 74 016)
Sex		
Men	85 850 (59.4)	44 585 (60.2)
Women	58 656 (40.6)	29 431 (39.8)
Age of death, y		
65-69	20 387 (14.1)	11 135 (15.0)
70-74	24 623 (17.0)	12 659 (17.1)
75-79	33 789 (23.4)	15 704 (21.2)
≥80	65 707 (45.5)	34 518 (46.6)
Residential area		
Urban	58 325 (40.4)	29 616 (40.0)
Rural	86 181 (59.6)	44 400 (60.0)
Occupation		
White collar	7174 (5.0)	4378 (5.9)
Blue collar	32 743 (22.7)	15 757 (21.3)
Pink collar	4927 (3.4)	2944 (4.0)
Other	99 662 (69.0)	50 937 (68.8)
Marital status		
Married	62 845 (43.5)	31 783 (42.9)
Single	81 661 (56.5)	42 233 (57.1)
Educational level		
Low	75 965 (52.6)	36 766 (49.7)
Middle	51 933 (35.9)	28 182 (38.1)
High	16 608 (11.5)	9068 (12.3)

**Table 2.** Distribution of the Study Population

Period	Death at home, No./total No. (%) (N = 218 522)				
	Cancer deaths (n = 207 459)			Dementia deaths (n = 11 063)	
	Yes	No	Yes		No
Preintervention period (February 2018 to August 2020)	11 837/136 390 (8.7)	124 553/136 390 (91.3)	2351/8116 (29.0)		5765/8116 (71.0)
Postintervention period (September 2020 to December 2021)	9014/71 069 (12.7)	62 055/71 069 (87.3)	895/2947 (30.4)		2052/2947 (69.6)
Total	20 851/207 459 (10.1)	186 608/207 459 (89.9)	3246/11 063 (29.3)		7817/11 063 (70.7)

Table 3 presents the results of the segmented regression analysis used to assess the probability of death at home after adjusting for all covariates. The difference in level change between the case and control groups at the time of the intervention was estimated to be 1.245. In other words, immediately after the introduction of home-based hospice care, the rate of deaths at home was 1.245 times greater for cancer deaths than for dementia deaths (exp [β_6_] = 1.245 [95% CI, 1.030-1.504]; *P* = .02). However, no significant difference was observed between the case and control groups regarding the trend change after the intervention compared with that before the intervention.

**Table 3.** Parameter Estimates, SEs, and *P* Values From the Segmented Regression Models Assessing the Probability of Death at Home

Parameter	exp (β)^a^	exp (SE[β]) (95% CI)	*P* value
Intercept (β_0_)	0.371	1.052 (0.336-0.410)	<.001
Control pretrend (β_1_)	1.006	1.003 (1.001-1.012)	.03
Control postlevel change (β_2_)	1.068	1.096 (0.893-1.279)	.47
Control posttrend change (β_3_)	0.983	1.009 (0.965-1.000)	.05
Case-control prelevel difference (β_4_)	0.220	1.057 (0.197-0.245)	<.001
Case-control pretrend difference (β_5_)	1.003	1.003 (0.997-1.009)	.27
Case-control postlevel change difference (β_6_)	1.245	1.101 (1.030-1.504)	.02
Case-control trend change difference pretrend to posttrend (β_7_)	1.009	1.010 (0.990-1.028)	.35

Abbreviation: exp, exponential.

^a^ Calculated by statistically adjusting for all covariates.

Table 3 provides quantitative confirmation of the differences in level and trend changes through the calculation of parameter estimates. The Figure allows for the intuitive confirmation of the outcome trends after the intervention. During the preintervention period, the probability of death at home tended to increase for both patients with cancer and those without cancer. At the time of the introduction of the intervention, both groups showed noticeable interruptions and absolute level changes, especially in cancer-related deaths. After the intervention, the likelihood of dying at home due to dementia began to decrease, whereas the likelihood of dying at home due to cancer was estimated to remain steady or increase slightly.

**Figure.**
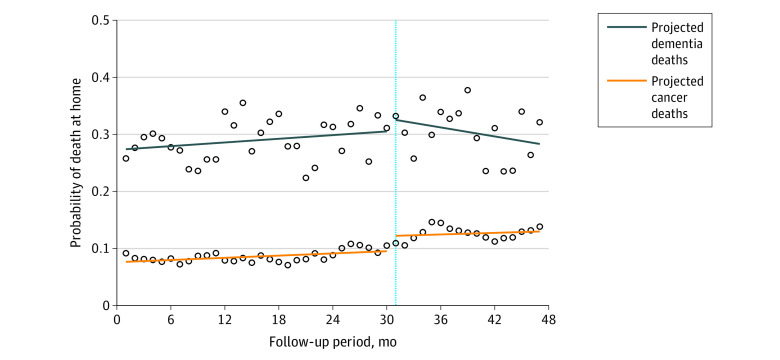
Estimated Trends in Probability of Death at Home The vertical dashed line indicates the start of the intervention on September 1, 2020. Circles indicate observed values.

We assumed that the preferred place of death would vary according to sociodemographic characteristics; therefore, we performed subgroup analyses stratified by residential area and educational level. As shown in Table 4, the difference in level change between cancer and dementia deaths, at intervention, was more pronounced for those living in rural areas (exp [β_6_] = 1.320 [95% CI, 1.118-1.558]; *P* = .001). In addition, a higher educational level was associated with a larger difference in immediate effect size due to home-based hospice practice (low educational level: exp [β_6_] = 1.205 [95% CI, 1.025-1.416]; *P* = .02; middle educational level: exp [β_6_] = 1.307 [95% CI, 0.987-1.730]; *P* = .06; high educational level: exp [β_6_] = 1.716 [95% CI, 0.932-3.159]; *P* = .08).

**Table 4.** Results of Subgroup Analysis Stratified by Residential Area and Educational Level

Variable	Death at home							
	exp (β_0_)	exp (β_1_)	exp (β_2_)	exp (β_3_)	exp (β_4_)	exp (β_5_)	exp (β_6_)	exp (β_7_)
**Residential area**								
Urban	0.242	1.002	1.277	0.978	0.298	1.007	1.074	1.011
*P* value	<.001	.66	.03	.04	<.001	.10	.55	.36
Rural	0.285	1.006	0.936	0.996	0.273	1.002	1.320	0.999
*P* value	<.001	.01	.40	.61	<.001	.35	.001	.86
**Educational level**								
Low	0.272	1.004	1.113	0.986	0.283	1.004	1.205	1.006
*P* value	<.001	.08	.15	.05	<.001	.11	.02	.45
Middle	0.269	1.006	0.936	0.987	0.271	1.003	1.307	1.004
*P* value	<.001	.17	.63	.35	<.001	.46	.06	.75
High	0.254	1.001	0.737	1.021	0.298	1.006	1.716	0.974
*P* value	<.001	.87	.31	.45	<.001	.53	.08	.36

Abbreviation: exp, exponential.

## Discussion

Since February 2018, Korea has been enforcing the Act on Hospice and Palliative Care and Decisions on Life-Sustaining Treatment for Patients at the End of Life, aimed at respecting the right to self-determination and improving quality of life for individuals at the end of life. Recently, there have been active discussions regarding the decision to discontinue life-sustaining treatment and die well, and various types of hospice services have been institutionally established. Hospice care in Korea was introduced in the order of hospital-based, home-based, and consultation services; the Korean Health Insurance Service covers all 3 types of services through health insurance. Nevertheless, until recently, most patients with terminal cancer use hospital-based hospices, with home-based and consultation services used only as supplemental care.^8^

In this context, the present retrospective cohort study investigated how the place of death of patients with cancer changed after the introduction of home-based hospice care in Korea and explored whether this intervention was being successfully implemented. The key findings of this study were as follows: in September 2020, when home-based hospice services began to be covered by health insurance in Korea, the probability of patients with cancer (who accounted for most hospice users) dying at home, rather than in a medical institution, increased markedly. However, dementia is not yet recognized as a disease requiring hospice services in Korea, and the probability of patients with dementia dying at home increased in the preintervention period but decreased in the postintervention period. Overall, the association between place of death of patients and the policy immediately after implementation showed a significant difference between groups with diseases eligible for hospice and those with diseases noneligible for hospice.

Most previous studies have examined the association of home-based hospice care with the economic burden on patients and their families. The studies revealed that home-based hospice services are effective in reducing medical expenses by preventing acute hospitalization, such as in an emergency department, and by managing symptoms competently at home.^16,17,18^ In addition, several studies have demonstrated that home-based hospice use improves both patient and caregiver satisfaction and quality of life.^19,20,21^ The studies suggested that home-based hospice care offers personalized and consistent care to patients and their families by providing the exclusive attention of a limited number of well-informed medical professionals. Several studies have investigated the determinants of the place of death for patients with terminal cancer receiving home hospice care,^22,23,24^ as well as the trends in their preferred and actual places of death.^25,26,27,28^ According to the findings reported, the presence of dedicated and capable caregivers, such as family members or trained hospice professionals, can be associated with the feasibility of home hospice care. Financial factors can also be associated with the place of death, and some patients may choose home hospice care because it is more cost-effective than inpatient care. Most patients with terminal illness have been found to prefer to die at home if given the choice; thus, home-based palliative care services that aim to provide patients with comprehensive medical, emotional, and practical support in their own homes are currently gaining popularity.

As such, interest in respecting the right to self-determination at the end of life and improving the quality of life of patients with terminal illness has increased. The extent and approach of hospice care coverage may differ from one country to another, and in certain countries, such as Korea, home-based hospice care services are covered by national health insurance. Although many patients with terminal illness wish to die comfortably with their families at home, to our knowledge, no study has evaluated whether the 2020 health insurance mandate for home-based hospice care services was associated with a change in the preferred place of death. Our results revealed that the probability of dying at home increased when home-based hospice care was covered by health insurance for patients with terminal cancer. The findings implied that, to enhance the quality of death of individuals with terminal illness and older adults, not only cancer and acute diseases but also more chronic and aging-related diseases, such as dementia and frailty, should be included as diseases eligible for home-based hospice care services.

### Limitations and Strengths

This study has certain limitations. First, the Causes of Death Statistics data that we analyzed included sociodemographic and mortality information on the total number of deaths in Korea; however, only the underlying cause of death was recorded, and there was no information on the use of medical care before death. Therefore, the recorded underlying cause of death codes could not represent the actual disease status of the patients, and there is a concern of misclassification problems because we could not take into account other contributing causes of death. In addition, because it was not possible to identify whether the deceased used home-based hospice care, using these data, the case and control groups were divided based on whether the patient died due to a hospice-eligible disease. Second, we used *ICD-10* codes to identify the study participants; however, the *ICD-10* codes themselves have limitations.^29,30,31^ The *ICD-10* codes are designed primarily for administrative purposes and may not provide comprehensive clinical details of patients. In addition, there is a concern of incomplete coding, which could result in the misclassification or underestimation of certain factors. Third, we had limited control over various additional nonofficial benefits and interventions provided to patients with terminal illness during the period in which the insurance coverage policy for home-based hospice care was implemented. Therefore, the associations could have possibly been overestimated. Fourth, although we attempted to adjust for potential confounders that could be associated with the change in the place of death, residual confounding effects from unmeasured variables could not be ruled out.

Nevertheless, the study has several strengths. The Causes of Death Statistics database that we analyzed contains very large-scale data, including the total number of deaths in Korea, which has the advantage of being applicable to the evaluation of the associations of medical practices with policies. In addition, we used the CITS design to assess the longitudinal effects of the interventions.^32^ Prior studies relied on a difference-in-differences study design, which compares only 2 time points to examine the net policy impact on outcomes,^33^ or a segmented regression with fewer than 10 time points.^34^ However, these approaches have limitations, because they do not adequately capture the baseline trends and changes. In contrast, our study used 47 time points, which enabled us to capture and analyze the trend changes more accurately over time.

## Conclusions

In this cohort study exploring the changes in the place of death of patients with terminal cancer after the insurance mandates went into effect for home-based hospice care in Korea, the probability of patients with cancer dying in their own homes increased after the intervention. To honor the autonomy of patients with terminal illness during the final stages of life and improve their quality of death, expansion of the coverage of home-based hospice care, including for other aging-related and chronic diseases, would be recommended.

## Notes

Supplement.
Data Sharing Statement

## References

[1] Ferlay J, Colombet M, Soerjomataram I, . Cancer statistics for the year 2020: an overview. Int J Cancer. 2021;149(4):778-789. doi:10.1002/ijc.33588 33818764

[2] Kirkova J, Davis MP, Walsh D, . Cancer symptom assessment instruments: a systematic review. J Clin Oncol. 2006;24(9):1459-1473. doi:10.1200/JCO.2005.02.8332 16549841

[3] Loke SS, Rau KM, Huang CF. Impact of combined hospice care on terminal cancer patients. J Palliat Med. 2011;14(6):683-687. doi:10.1089/jpm.2010.0331 21504306 PMC3107584

[4] Krakauer EL, Kwete X, Verguet S, . Palliative care and pain control. In: Jamison DT, Gelband H, Horton S, et al, eds. Disease Control Priorities: Improving Health and Reducing Poverty. 3rd ed. The International Bank for Reconstruction and Development/The World Bank; 2017. doi:10.1596/978-1-4648-0527-1_ch12 30212058

[5] Saunders C. Hospice care. Am J Med. 1978;65(5):726-728. doi:10.1016/0002-9343(78)90789-1 81612

[6] World Health Organization. Why Palliative Care Is an Essential Function of Primary Health Care. World Health Organization; 2018.

[7] World Health Organization. Quality Health Services and Palliative Care: Practical Approaches and Resources to Support Policy, Strategy and Practice. World Health Organization; 2021.

[8] Kim K, Park B, Gu B, Nam EJ, Kye SH, Choi JY. The National Hospice and Palliative Care registry in Korea. Epidemiol Health. 2022;44:e2022079. doi:10.4178/epih.e2022079 36177979 PMC9943630

[9] Penrod JD, Deb P, Luhrs C, . Cost and utilization outcomes of patients receiving hospital-based palliative care consultation. J Palliat Med. 2006;9(4):855-860. doi:10.1089/jpm.2006.9.855 16910799

[10] Penrod JD, Deb P, Dellenbaugh C, . Hospital-based palliative care consultation: effects on hospital cost. J Palliat Med. 2010;13(8):973-979. doi:10.1089/jpm.2010.0038 20642361

[11] Kim JH, Yoo KB, Lee Y. Development and validation of the Korea Dementia Comorbidity Index (KDCI): a nationwide population-based cohort study from 2002 to 2013. Arch Gerontol Geriatr. 2017;72:195-200. doi:10.1016/j.archger.2017.06.001 28709115

[12] Kim YJ, Han JW, So YS, Seo JY, Kim KY, Kim KW. Prevalence and trends of dementia in Korea: a systematic review and meta-analysis. J Korean Med Sci. 2014;29(7):903-912. doi:10.3346/jkms.2014.29.7.903 25045221 PMC4101777

[13] Shon C, Yoon H. Health-economic burden of dementia in South Korea. BMC Geriatr. 2021;21(1):549. doi:10.1186/s12877-021-02526-x 34645415 PMC8515696

[14] Somers MA, Zhu P, Jacob R, Bloom H. The Validity and Precision of the Comparative Interrupted Time Series Design and the Difference-in-Difference Design in Educational Evaluation. MDRC; 2013.

[15] Wagner AK, Soumerai SB, Zhang F, Ross-Degnan D. Segmented regression analysis of interrupted time series studies in medication use research. J Clin Pharm Ther. 2002;27(4):299-309. doi:10.1046/j.1365-2710.2002.00430.x 12174032

[16] Gomes B, Calanzani N, Curiale V, McCrone P, Higginson IJ, de Brito M. Effectiveness and cost-effectiveness of home palliative care services for adults with advanced illness and their caregivers. Cochrane Database Syst Rev. 2013;2013(6):CD007760. doi:10.1002/14651858.CD007760.pub2 23744578 PMC4473359

[17] McCaffrey N, Agar M, Harlum J, Karnon J, Currow D, Eckermann S. Is home-based palliative care cost-effective? an economic evaluation of the Palliative Care Extended Packages at Home (PEACH) pilot. BMJ Support Palliat Care. 2013;3(4):431-435. doi:10.1136/bmjspcare-2012-000361 24950523

[18] Lee J. *Study on the Role of the Parish Community for the Hospice Home Care*. Dissertation. Catholic University; 2002.

[19] McMillan SC, Small BJ. Symptom distress and quality of life in patients with cancer newly admitted to hospice home care. Oncol Nurs Forum. 2002;29(10):1421-1428. doi:10.1188/02.ONF.1421-1428 12432413

[20] Tang WR. Hospice family caregivers’ quality of life. J Clin Nurs. 2009;18(18):2563-2572. doi:10.1111/j.1365-2702.2008.02753.x 19538564

[21] Grande GE, Farquhar MC, Barclay SI, Todd CJ. Caregiver bereavement outcome: relationship with hospice at home, satisfaction with care, and home death. J Palliat Care. 2004;20(2):69-77. doi:10.1177/082585970402000202 15332470

[22] Tang ST, McCorkle R. Determinants of place of death for terminal cancer patients. Cancer Invest. 2001;19(2):165-180. doi:10.1081/CNV-100000151 11296621

[23] Moinpour CM, Polissar L. Factors affecting place of death of hospice and non-hospice cancer patients. Am J Public Health. 1989;79(11):1549-1551. doi:10.2105/AJPH.79.11.1549 2817170 PMC1349812

[24] Fukui S, Kawagoe H, Masako S, Noriko N, Hiroko N, Toshie M. Determinants of the place of death among terminally ill cancer patients under home hospice care in Japan. Palliat Med. 2003;17(5):445-453. doi:10.1191/0269216303pm782oa 12882263

[25] Neergaard MA, Jensen AB, Sondergaard J, Sokolowski I, Olesen F, Vedsted P. Preference for place-of-death among terminally ill cancer patients in Denmark. Scand J Caring Sci. 2011;25(4):627-636. doi:10.1111/j.1471-6712.2011.00870.x 21362004

[26] De Conno F, Caraceni A, Groff L, . Effect of home care on the place of death of advanced cancer patients. Eur J Cancer. 1996;32A(7):1142-1147. doi:10.1016/0959-8049(96)00036-6 8758244

[27] Beccaro M, Costantini M, Giorgi Rossi P, Miccinesi G, Grimaldi M, Bruzzi P; ISDOC Study Group. Actual and preferred place of death of cancer patients: results from the Italian Survey of the Dying of Cancer (ISDOC). J Epidemiol Community Health. 2006;60(5):412-416. doi:10.1136/jech.2005.043646 16614331 PMC2563975

[28] Dasch B, Blum K, Gude P, Bausewein C. Place of death: trends over the course of a decade: a population-based study of death certificates from the years 2001 and 2011. Dtsch Arztebl Int. 2015;112(29-30):496-504. doi:10.3238/arztebl.2015.0496 26249252 PMC4555061

[29] Surján G. Questions on validity of *International Classification of Diseases*–coded diagnoses. Int J Med Inform. 1999;54(2):77-95. doi:10.1016/S1386-5056(98)00171-3 10219948

[30] Stausberg J, Lehmann N, Kaczmarek D, Stein M. Reliability of diagnoses coding with *ICD-10*. Int J Med Inform. 2008;77(1):50-57. doi:10.1016/j.ijmedinf.2006.11.005 17185030

[31] Quan H, Li B, Saunders LD, ; IMECCHI Investigators. Assessing validity of *ICD-9-CM* and *ICD-10* administrative data in recording clinical conditions in a unique dually coded database. Health Serv Res. 2008;43(4):1424-1441. doi:10.1111/j.1475-6773.2007.00822.x 18756617 PMC2517283

[32] Cook TD, Campbell DT, Shadish W. Experimental and Quasi-Experimental Designs for Generalized Causal Inference. Houghton Mifflin Boston; 2002.

[33] Dimick JB, Ryan AM. Methods for evaluating changes in health care policy: the difference-in-differences approach. JAMA. 2014;312(22):2401-2402. doi:10.1001/jama.2014.16153 25490331

[34] Kessler D, McClellan M. Do doctors practice defensive medicine? Q J Econ. 1996;111(2):353-390. doi:10.2307/2946682

