# Supplementary material for: Changes in the Place of Death of Patients With Cancer After the Introduction of Insurance-Covered, Home-Based Hospice Care in Korea
Source: JAMA Netw Open. 2023 Nov 6;6(11):e2341422. doi: 10.1001/jamanetworkopen.2023.41422 (PMC10628724; doi:10.1001/jamanetworkopen.2023.41422)
Supplement: Supplement. — Data Sharing Statement [file jamanetwopen-e2341422-s001.pdf]

Data Sharing Statement

Yun. Changes in the Place of Death of Patients With Cancer After the Introduction of Insurance-Covered, Home-Based Hospice Care in Korea. *JAMA Netw Open.* Published November 06, 2023. doi:10.1001/jamanetworkopen.2023.41422

Data
Data available: Yes
Data types: Deidentified participant data
How to access data: The Cause of Death Statistics is publicly accessible and can be shared through application on the website of Korea Statistics Promotion Institute (https://mdis.kostat.go.kr/).
When available: With publication

Supporting Documents
Document types: None

Additional Information
Who can access the data: Anyone who wants to use the data is available.
Types of analyses: Any analysis using data is available.
Mechanisms of data availability: N/A
